# Retrospective evaluation of prognosis and survival with various immunosuppressants in 82 dogs diagnosed with meningoencephalitis of unknown etiology (2010–2021)

**DOI:** 10.1186/s12917-023-03800-3

**Published:** 2023-12-12

**Authors:** So-Hee Kim, Ye-In Oh, Su-Min Park, Ju Hyun An, Tae-Hee Kim, Sung-Soo Kim, Jae-Gon Ah, Kyoung-Won Seo, Hwa-Young Youn

**Affiliations:** 1 Laboratory of Veterinary Internal Medicine, Department of Veterinary Clinical Science, College of Veterinary Medicine, Seoul National University, Seoul, 00826 Republic of Korea; 2 Department of Veterinary Internal Medicine, College of Veterinary Medicine, Kyungpook National University, Daegu, 41566 Republic of Korea; 3 Department of Veterinary Emergency and Critical Care Medicine and Institute of Veterinary Science, College of Veterinary Medicine, Kangwon National University, Chuncheon-si, 24341 Republic of Korea; 4 VIP Animal Medical Center KR, Seoul, 02830 Republic of Korea

**Keywords:** Adjunctive immunosuppressant, Dog, Leflunomide, Meningoencephalomyelitis of unknown etiology (MUE)

## Abstract

**Background:**

Meningoencephalomyelitis of unknown etiology (MUE) is a comprehensive term for non-infectious inflammatory brain diseases of the central nervous system (CNS) caused by abnormal autoimmune responses. This study aims to compare the differences in survival and clinical response of MUE according to the adjuvant immunosuppressant use. Medical records of 82 dogs diagnosed with MUE were reviewed retrospectively.

**Results:**

The overall survival time was 769 days (range 14–2687 days). The median survival time for each adjunctive was: leflunomide 1035 days (range 126–2163 days), mycophenolate mofetil 865 days (range 39–2191 days), cyclosporin 441 days (range 11–2176 days), cytosine arabinoside 754 days (range 6–1898 days) and a combination of mycophenolate mofetil and cytosine arabinoside 132 days (range 23–1227 days). There was no significant difference in the incidence rate of adverse events according to the immunosuppressants, but moderate to severe anemia was confirmed in 3 patients (18.7%) in the leflunomide group.

**Conclusions:**

The survival time and response rate of MUE dogs differed depending on which adjunctive immunosuppressants were used. Leflunomide showed a long survival time and a relatively good response rate in dogs with MUE. However, a large-scale further study with standardized doses of immunosuppressants and supportive treatment and constant monitoring interval is needed.

## Background

Meningoencephalomyelitis of unknown etiology (MUE) is a comprehensive term for non-infectious inflammatory brain diseases of the central nervous system (CNS) and is histologically divided into granulomatous meningoencephalomyelitis, necrotizing meningoencephalitis, and necrotizing leukoencephalitis [1]. Because a diagnosis can only be confirmed through post-mortem histopathology, clinical diagnosis is made by combining the patient’s symptoms, clinical signs, neurological examination, magnetic resonance imaging (MRI)/computed tomography results, and cerebrospinal fluid (CSF) analysis [2].

There is no clear mechanism identified for the pathophysiology of MUE, but it is known to be caused by abnormal immune responses to CNS components [3]. As such, immunosuppressive doses of glucocorticoids have been used as a standard treatment method. Using adjunctive immunosuppressants has been increased in order to prevent recurrence of symptoms in the process of reducing glucocorticoids and to reduce the systemic side effects of glucocorticoids such as polydipsia, polyuria, steroid-induced hepatopathy, increased likelihood of infection, delayed wound healing, calcinosis cutis, and muscle weakness [2, 4, 5]. Although there are few reports directly comparing glucocorticoid monotherapy with the combination of adjunctive immunosuppressants and glucocorticoid, it has shown that adjunctive immunosuppressants such as cytarabine, cyclosporin, azathioprine, mycophenolate mofetil (MMF) have good effects and can increase the survival time in MUE patients [6–13]. However, because the sample size was relatively small, comparison between treatment groups has been limited. In particular, leflunomide has been shown to have a therapeutic effect on several autoimmune diseases in dogs, there is only one report of leflunomide used for MUE in three dogs; therefore, data regarding the efficacy and safety of leflunomide are insufficient [14, 15]. This study aims to compare the differences in survival and clinical response of MUE according to the use of adjuvant immunosuppressant use. In addition, the complications that may appear according to the use of immunosuppressants were investigated.

## Results

### Signalments and clinical signs in affected dogs

A total of 82 dogs were included in this retrospective study. The breed distribution was as follows: Maltese (n = 40), Chihuahua (n = 14), Pomeranian (n = 10), Yorkshire Terrier (n = 5), Miniature Poodle (n = 4), mixed breed (n = 2), and one each of Cocker spaniel, Golden Retriever, Boston Terrier, French Bulldog, Shih-Tzu, Spitz, and Silky Terrier. Forty-three dogs (52%) were female (intact, n = 20; spayed, n = 23), and 39 dogs (48%) were male (intact, n = 8; castrated, n = 31). Bodyweights at the time of diagnosis ranged from 0.83 to 26.97 kg (median = 3 kg, mean = 3.658 kg) and the range of time from symptom onset to hospital presentation was 0 days to 1,254 days (median = 6 days, mean = 52.73 days).

The neurological symptoms that were identified during the initial visit were as follows: seizure (n = 40), blindness (n = 24), circling (n = 23), head turning (n = 18), nystagmus (n = 14), head tilting (n = 12), ataxia (n = 10), behavioral changes (n = 10), hemiparesis (n = 10), mental status change (n = 9), tremor (n = 8), paraparesis (n = 7), gait abnormalities (n = 6), upper motor neuron paralysis (n = 4), breathing abnormalities (n = 4), tetraparesis (n = 3), proprioceptive deficits (n = 3), narcolepsy-cataplexy (n = 2), fever (n = 2), hypothermia (n = 2), cerebellar ataxia (n = 1), kyphosis (n = 1), pain (n = 1), head pressing (n = 1), and strabismus (n = 1) (Table 1).

**Table 1:** Neurologic symptoms during the initial visit

Neurological symptoms	Number of dogs	Percent (%)
Seizure	40	49%
Blindness	24	29%
Circling	23	28%
Head turn	18	22%
Cluster seizure	14	17%
Nystagmus	14	17%
Head tilt	12	15%
Ataxia	10	12%
Behavioral change	10	12%
Hemiparesis	10	12%
Mental change	9	11%
Tremor	8	10%
Paraparesis	7	9%
Gait abnormalities	6	7%
UMN paralysis	4	5%
Tetraparesis	3	4%
Proprioceptive deficits	3	4%
Narcolepsy-cataplexy	2	2%
Breathing abnormalities	2	2%
Breathing abnormalities	2	2%
Febrile	2	2%
Hypothermia	2	2%
Cerebellar atazia	1	1%
Kyphosis	1	1%
Painful reaction	1	1%
Proprioceptive deficit	1	1%
Head pressing	1	1%
Strabismus	1	1%
Breathing abnormalities	1	1%

### MRI findings

MRI scans were performed in all dogs; 14 dogs had focal lesions, and 68 dogs had multifocal lesions. The affected neurological sites were in the forebrain (49 dogs), brainstem (two dogs), and spinal cord (two dogs), while the remaining 29 dogs had lesions at more than two sites in the forebrain, brainstem, cerebellum, and spinal cord. On the MRI, caudal occipital malformation syndrome (COMS) was identified in 48 dogs, syringomyelia in 20 dogs, hydrocephalus in 14 dogs, and ventriculomegaly in 24 dogs.

### CSF analysis

CSF results were analyzed from 44 dogs. Elevated total protein levels or pleocytosis in cytology in CSF analysis could be evidence of MUE, but patients whose CSF test results were within the normal range or who failed to proceed with CSF analysis due to cerebellar herniation or systemic instability were not excluded from the population since previous studies have a history of confirming inflammatory CNS diseases in a biopsy, even though CSF test results were normal [4, 16–19]. Total nucleated cell count (TNCC) was identified in 40 dogs, and the median TNCC was 10 cells/µL (range: 0–5733 cells/µL). Total protein tests were conducted in 39 dogs using urinary reagent strips, of which 30 were negative (< 30 mg/L), 8 dogs were confirmed to have 1 positive finding (30–100 mg/dL), and only 1 dog showed 2 positive results (> 100 mg/dL) [20]. Of the 43 dogs subjected to cytologic examination, 27 dogs had mononuclear pleocytosis, and the cell type was either lymphocyte dominant (n = 11, 41%), monocyte dominant (n = 6, 22%), or mixed cell type (n = 10, 37%). PCR results were negative for all dogs, and no bacteria were detected in the bacterial culture test.

### Treatment

Dogs diagnosed with MUE were treated with PDS alone or PDS in combination with adjunctive immunomodulating drugs. The dose of PDS was reduced by 25% every 2 to 4 weeks when the dogs showed improvement or when side effects from taking PDS were intolerable. Adjunctive immunomodulating drugs were reduced when side effects were suspected, or there was no recurrence of neurological symptoms, even after PDS was reduced. The initial doses of each drug were as follows: MMF (median 12 mg/kg, range 10–20 mg/kg PO, q12h), cyclosporine (median 6.4 mg/kg, range 5–12.5 mg/kg PO, q24h or median 8.5 mg/kg, range 4–15 mg/kg PO, q12h), and leflunomide (2 mg/kg PO, q12h or median 4 mg/kg, range 3–4 mg/kg PO, q24h). Cytosine arabinoside (cytarabine) was injected subcutaneously (50 mg/m^2^ SC, q12h, for 2 days) or intravenously (25 mg/m^2^/h, for 8 h) at 3 to 4-week intervals [6]. The type of adjuvant immunosuppressants was determined according to the preference of the clinicians or the financial cause of the clients. In 17 dogs, the adjunctive immunosuppressants were changed due to lack of treatment response, if symptoms recurred/worsened, or for financial reasons, and the adjunctive immunosuppressants were changed again in 3 out of 17 dogs. Those patients that were changed the agents were included in duplicate in each group. Mycophenolate mofetil (MMF) (n = 26), cytarabine (n = 20), cyclosporin (n = 17), or leflunomide (n = 16) were used as adjunctive agents; in some dogs, a combination of MMF + cytarabine (n = 9), MMF + cyclosporin (n = 2), or cyclosporin + cytarabine (n = 1) was used.

The improvement of neurological symptoms was evaluated at each visit based on the subjective evaluation of the owners and the neurological examination result by the attending clinicians. The treatment response was classified as a complete response (CR) if the neurological signs completely disappeared and did not recur during the follow-up period, a partial response (PR) if symptoms decreased by more than 50% but remained, or if symptoms recurred within one month of stating the agents; or no response (NR) when symptoms did not improve or worsened.

### Supportive treatment

Anticonvulsants were prescribed to patients whose seizures were not controlled by immunosuppressive treatments. Phenobarbital (2 mg/kg PO, q12h–5 mg/kg PO, q8h), levetiracetam (10 mg/kg PO, q12h–20 mg/kg PO, q8h), zonisamide (5–10 mg/kg PO, q12h), and potassium bromide [KBr] (20 mg/kg PO, q24h or 15–20 mg/kg PO, q12h) have shown to be effective. For each agent, 3 out of 11 patients (27%) in the PDS monotherapy group, 10 out 26 patients (38%) in the MMF group, and 9 out of 18 patients (50%) in the cyclosporin group, 8 out 16 patients (50%) in the leflunomide group and 11 out of 19 (58%) patients in cytarabine group were prescribed anticonvulsants. In triple immunosuppressive treatment groups, 8 out of 9 patients in combination of MMF + cytarabine (89%) group and one patient (100%) in the cyclosporin + cytarabine group were prescribed anticonvulsants, and two patients (0%) in MMF + cyclosporin group didn’t take the anticonvulsants.

If the dogs showed pain responses or were concerned about pain due to accompanying syringomyelia, neurological painkillers like gabapentin (7.5 mg/kg PO, q12h–15 mg/kg PO, q8h), pregabalin (2 mg/kg or 4 mg/kg PO, q12h) or methocarbamol (20–25 mg/kg PO, q12h) were used. Gastrointestinal protectants were used to prevent gastrointestinal malaise by PDS or to treat adverse gastrointestinal events due to the long-term use of PDS or other immunomodulatory agents. In such cases, famotidine (0.5 mg/kg PO, q12h), omeprazole (0.5–1 mg/kg PO, q12h), esomeprazole (1 mg/kg PO, q12h), or misoprostol (5 µg/kg PO, q12h or q24h) were prescribed. Silymarin (10–20 mg/kg PO, q12h) and ursodeoxycholic acid (5–20 mg/kg PO, q12h) were used as liver protectants to prevent steroid-induced hepatopathy or to lower liver enzyme elevation.

### Analysis of survival time after adjunctive immunosuppressant treatment

**Fig. 1:**
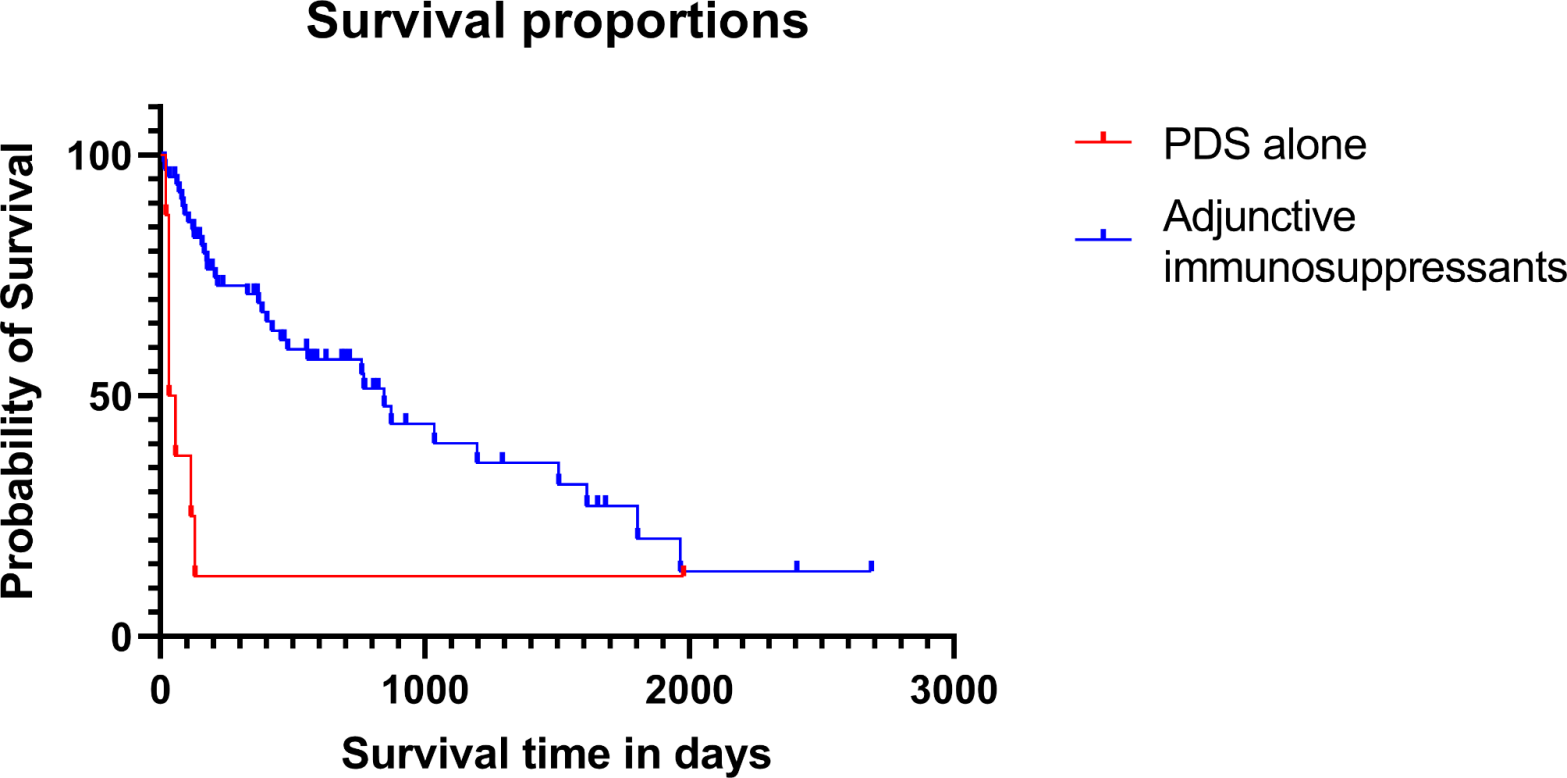
Kaplan-Meier graph for comparing survival time of prednisolone (PDS) alone and PDS with adjunctive immunosuppressants. The median survival time of the PDS alone group (n = 8) was 42 days (range 21-1978 days) while the median survival time of using PDS with adjunctive immunosuppressants (n = 65) was 846 days (range 14-2678 days). Dogs that were alive at the end of the study or died for reasons other than MUE were censored. Censored points are marked with dots. There was a statistical difference between them (*p* = 0.005)

Survival and time of death were followed-up in 73 of the 82 dogs. Forty-seven dogs had died and the causes of death were either neurological in nature (n = 37), side effects from prolonged use of PDS(n = 5), tumors such as splenic hemangiosarcoma, leukemia and mammary gland carcinoma (n = 3), post-surgery sepsis (n = 1), or ingestion of a foreign body (n = 1). The overall survival time of the 73 dogs was 769 days (range: 14–2,687 days). Eleven dogs were treated with prednisolone (PDS) alone, and the remaining 71 dogs were treated with PDS and other adjunctive immunosuppressants. The median survival time (MST) of the 8 patients treated with PDS alone was 42 days (range: 21–1,978 days), while that of the 65 patients treated with adjunctive immunosuppressants was 846 days (range: 14–2,687 days). The MST of adjunctive immunosuppressants group was significantly higher comparing with PDS monotherapy group (*p* = 0.005) (Fig. 1).

**Fig. 2:**
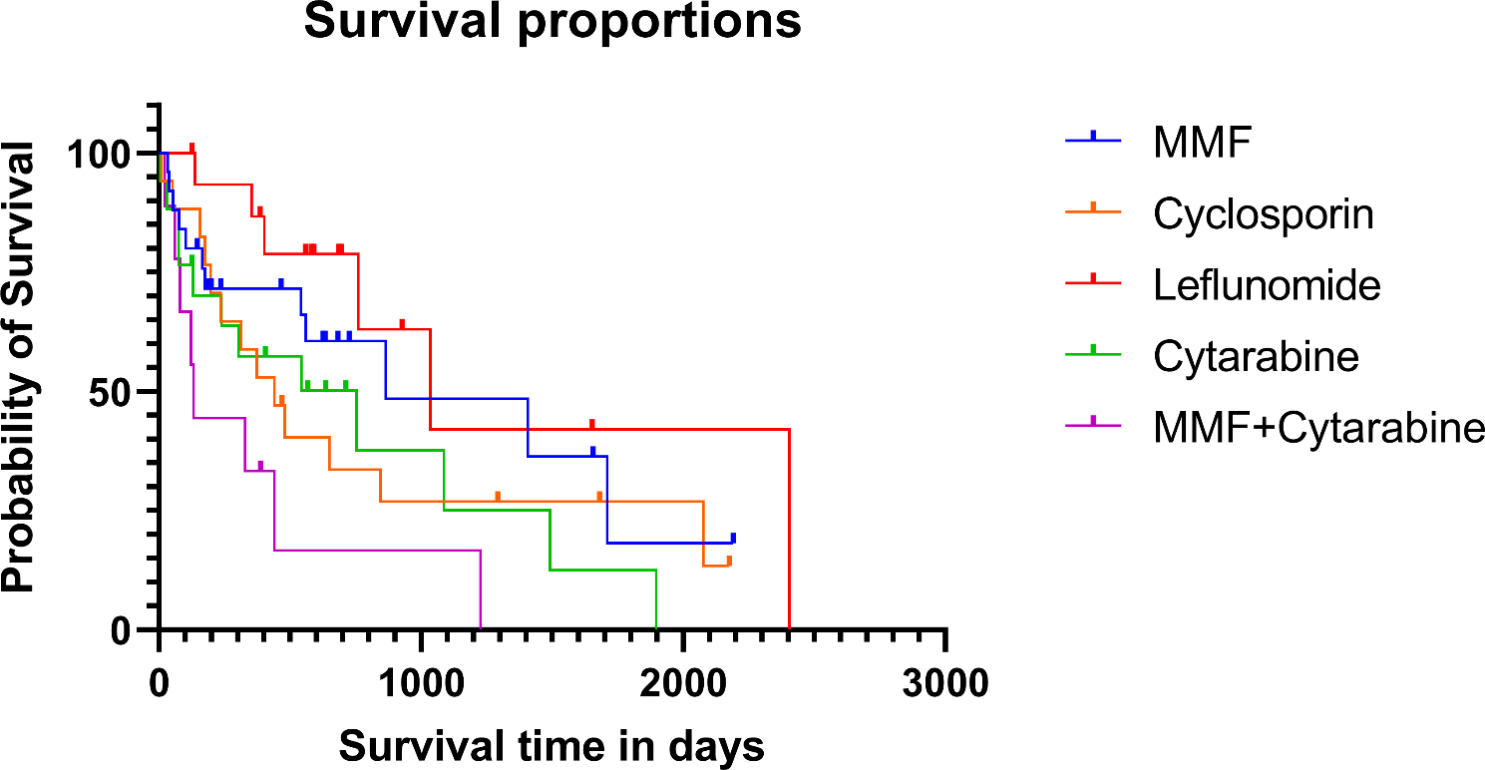
Kaplan-Meier graph for comparing survival time according to which adjunctive immunosuppressants were used. The median survival time was 1035 days (range 126–2163 days) in the leflunomide (n = 16) group, 865 days (range 39-2191 days) in the mycophenolate mofetil group (MMF) (n = 26), 441 days (range 11-2176 days) in the cyclosporin group (n = 18), 754 days (range 6-1898 days) in cytarabine group (n = 19), and 132 days (range 23 ~ 1227 days) in the combination of MMF and cytarabine group (n = 9). Dogs that were alive at the end of the study or died for reasons other than MUE were censored. Censored points are marked with dots. There was a significant difference in the survival time between each treatment group (*p* = 0.016)

The survival times from the start of drug administration to death or end of study were compared for each adjunctive immunosuppressant. The leflunomide group had the longest MST of 1,035 days (range: 126–2,163 days), and the group using a combination of MMF + cytarabine had the shortest MST of 132 days (range: 23–1,227 days). The MST was 865 days (range, 39–2191 days) in the MMF group, 441 days (range: 11–2,176 days) in the cyclosporin group, and 754 days (range: 6–1898 days) in cytarabine group. The log rank test from the Kaplan Meier survival analysis revealed a significant difference (*p* = 0.016) between each treatment groups (Table 2) (Fig. 2). In the MMF + cyclosporin group, one dog died from MUE after 63 days of treatment, and the other survived until the end of the study with 570 days of follow-up period. A dog who was treated with cyclosporin + cytarabine died after 761 days of treatment due to worsening of neurological symptoms.

**Table 2:** Survival time and treatment response according to the agents

Treatment (n)	Median Survival time (day)	Response (%)
PDS alone (11)	42	CR = 3 (27%)
	(range 21–1978)	PR = 3 (27%)
		NR = 5 (45%)
MMF (26)	865	CR = 5 (19%)
	(range 39–2191)	PR = 17 (65%)
		NR = 4 (15%)
Cyclosporin (18)	441	CR = 5 (19%)
	(range 11–2176)	PR = 5 (28%)
		NR = 8 (44%)
Leflunomide (16)	1035	CR = 2 (13%)
	(range 126–2163)	PR = 11 (69%)
		NR = 3 (19%)
Cytarabine (19)	754	CR = 5 (26%)
	(range 6–1898)	PR = 4 (21%)
		NR = 10 (53%)
MMF + Cytarabine (9)	132	PR = 5 (56%)
	(range 23–1227)	NR = 4 (44%)
MMF + Cyclosporin (2)	–	PR = 1 (50%)
		NR = 1 (50%)
Cyclosporin + Cytrabine (1)	–	NR = 1 (100%)

MMF: mycophenolate mofetil; Cytarabine: cytosine arabinoside; CR: complete response; PR: partial response; and NR: no response

### Differences in treatment response by adjunctive immunosuppressants

**Fig. 3:**
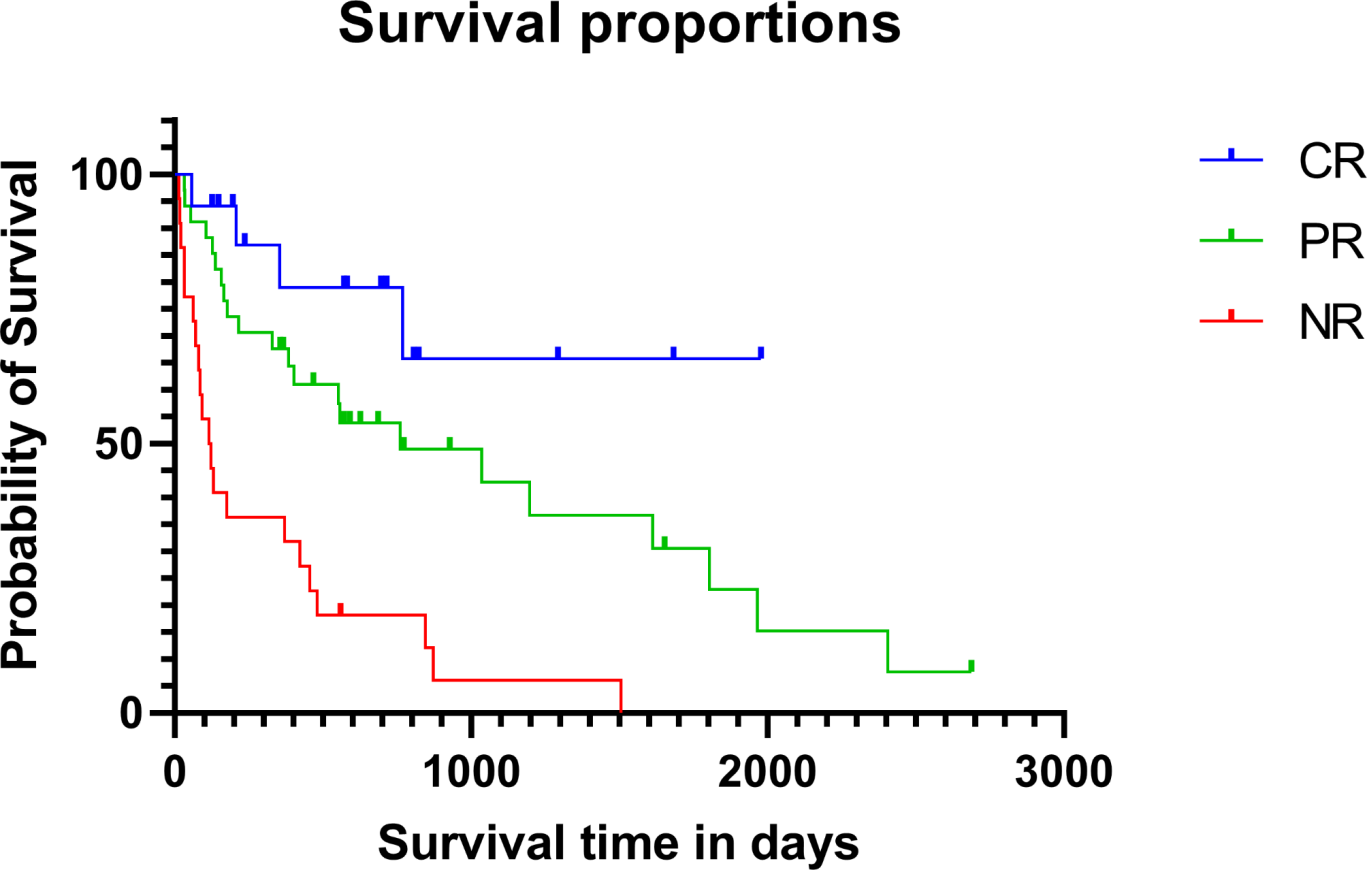
Kaplan-Meier graph for 73 dogs in the complete response (CR) (n = 17, 23%), partial response (PR) (n = 34, 47%), and no response (NR) (n = 22, 30%) groups. The median survival time (MST) of the CR group could not be calculated because 68% of the patients were alive at the end of the study. The MST was 761 days (range 32-2687 days) in the PR group, 118.5 days (range 14-1505 days). Dogs that were alive at the end of the study or died for reasons other than MUE were censored. Censored points are marked with dots. There was a significant difference in the survival time between each treatment group (*p* < 0.0001)

Evaluation of the subjects’ overall treatment response was based on the response at the end of the follow-up period. The median follow-up period from date of visit hospital to the date of last visit or death or end of study is 220.5 days (range: 17–2,687 days). Of the 82 cases, 19 cases were evaluated as complete response (CR) (23%), 37 cases as partial response (PR) (45%), and 26 cases as no response (NR) (32%). Thirteen cases of CR were alive at the end of the study (68%), so it was difficult to estimate the median survival time, but the survival time ranged from 57 to 1,978 days, and they had a longer overall survival time compared to the PR and NR groups. The MST of the PR group was 761 days (range: 32–2,687 days), and the MST of the NR group was 118.5 days (range: 14–1,505 days). The log rank test from the Kaplan Meier analysis revealed a significant difference (*p* < 0.0001) among each treatment groups (Fig. 3).

Treatment response to each adjunctive immunosuppressant was assessed at the time of use. MMF had the best overall response (CR + PR) rate with 84%, followed by leflunomide with 82%. The overall response rate to PDS monotherapy was 54%, and both cyclosporine and cytarabine had a 47% overall response rate. Among the 2 dogs treated with MMF + cyclosporin, one dog showed PR, and the other showed NR, so the response rate was evaluated to be 50%. One dog treated with cyclosporin + cytarabine was evaluated as NR (Table 2).

### Other factors affecting the survival time

It has been reported that the number of lesions or coexistent lesions on MRI, breed or control of seizure could affect survival time or relapse [2, 4, 21, 22]. Considering previous studies, we evaluated which other factors affected the patients’ prognosis in 73 dogs whose survivals were followed. The association between survival time and various factors was analyzed. Whether each of the variables influenced the survival time individually, the dogs with seizure had a 2.311-fold higher hazard ratio than that of the dog without seizure (*p* = 0.011, 95% CI = 0.2179–1.517). There is no significant association for other variables.

**Fig. 4:**
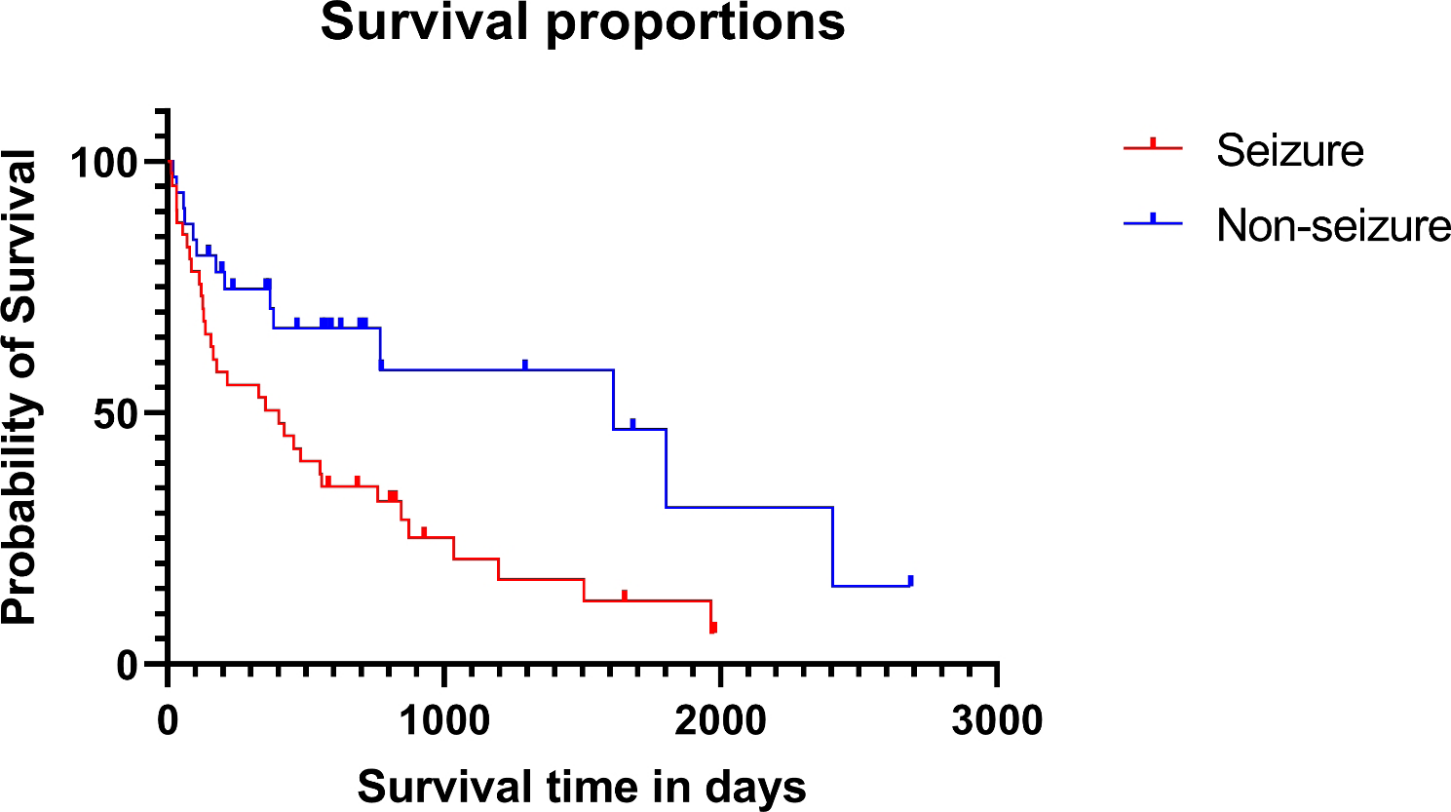
Kaplan-Meier graph for 73 dogs in the seizure (n = 41, 56%) or non-seizure group (n = 32, 44%). The median survival time of the seizure group was 403 days (range 14-1978 days) in the seizure group, and 1612 days (21-2687 days) in the non-seizure group. Dogs that were alive at the end of the study or died for reasons other than MUE were censored. Censored points are marked with dots. There was a significant difference in the survival time between each treatment group (*p* = 0.009)

In the univariate multiple Cox regression analysis, whether each factor affects the survival time when the factors are present simultaneously was investigated. The presence of seizures upon admission was related to the short survival time with 2.652-fold hazard ratio (*p* = 0.012, 95% CI 1.268–5.798). When comparing the Kaplan Meier survival curve, the MST of dogs with seizure symptoms upon admission was 403 days (range: 14–1978 days), and the MST of dogs without seizures was 1,612 days (range: 21–2687 days) (Fig. 4). The other factors investigated did not have a significant association with survival time.

### Adverse events related to adjunctive immunosuppressant use

In dogs treated with MMF, acute adverse events were confirmed in 6 out of 26 dogs (23%), including gastrointestinal complications (n = 3), skin infection (n = 1), and lethargy (n = 1). One dog had a hematological abnormality (grade 1 anemia; packed cell volume [PCV], 32.7%; reference range 37.1–57%). Vomiting and diarrhea symptoms improved after supportive care, such as switching to a low-fat diet or taking probiotics. A dog with grade 1 anemia (PCV, 32.7%; reference range 37.1–57%) was confirmed 3 weeks after treatment administration but returned to a normal range after 7 days.

Adverse events were identified in 10 out of 18 dogs (55.5%) in the cyclosporin group, including gastrointestinal complications (n = 7) and skin infection (n = 1). Two dogs had severe gastrointestinal problems, one dog improved after discontinuation of cyclosporin, and the other had vomiting, melena, and diarrhea after increasing the dose from 5 mg/kg to 10 mg/kg once a day and died 7 days after the increase. Hematologic abnormalities were identified in two dogs; 1 dog had a grade 1 anemia (PCV 30.6%) one month after treatment administration, and the other had grade 1 thrombocytopenia (platelet 132,000 cells/µL; reference range: 143,000–400,000), 10 days after treatment administration. One subject with mild anemia showed improvement after a reduction in PDS dose, and thrombocytopenia had improved at the next visit without requiring any additional treatment.

The leflunomide group had adverse events in 6 of 16 cases, including vomiting (n = 2), within 2 weeks of treatment initiation. Anemia was confirmed in four dogs: one had grade 1 anemia (PCV 35.3%), two dogs had grade 2 anemia (PCV 26% and 24.3%, respectively), and one dog had severe grade 4 anemia (PCV 12.6%). Among dogs with moderate anemia, one (PCV 26%) improved spontaneously without any other treatment, and one (PCV 24.3%) improved after discontinuation of leflunomide. The subject with grade 4 anemia showed severe anemia with lethargy on the 103rd day of drug administration and was confirmed as having non-regenerative anemia with a reticulocyte count of 2,200 cells/µL.

The cytarabine group showed adverse signs in 7 of 19 dogs. Five dogs had gastrointestinal complications such as hematochezia (n = 3), diarrhea (n = 2), and one of them also had skin infection. One dog had lethargy and the other had urinary tract infection. Of the seven dogs with clinical signs, four dogs also showed hematological abnormalities. Upon Complete blood count (CBC) examination, grade 1 anemia was confirmed in two dogs (PCV 33% and 34.2%, respectively) and grade 2 anemia in two dogs (PCV 28% and 28.3%, respectively). In the group using MMF + cytarabine, six dogs showed adverse events, including gastrointestinal complications (n = 2), skin infection (n = 1), and anemia (n = 3). Two dogs had grade 1 anemia (PCV 30.6% and 30.8%, respectively) and one dog had grade 2 anemia (PCV 29.1%). In the group using MMF + cyclosporine, one out of two dog showed vomiting and anorexia 9 days after treatment initiation, which improved after supportive care. No adverse events were observed in the two dogs that received cyclosporine and cytarabine (Table 3). Hepatoxicity was assessed in 43 dogs, renal toxicity was assessed in 36 dogs, and no significant increases were observed compared to pre-treatment levels in any adjunctive immunosuppressive therapy groups.

**Table 3:** Adverse events according to the adjunctive immunosuppressants

Treatment (n)	Adverse events (n)
MMF (26)	23% (6)
	GI signs (3), Infection (1) lethargy (1), Anemia (1)
Cyclosporin (18)	55.5% (10)
	GI signs (7), Infection (1), Anemia (1), Thrombocytopenia (1)
Leflunomide (16)	37.5% (6)
	GI signs (2), Anemia (4)
Cytarabine (19)	36.8% (7)
	GI signs (5), Lethargy (1), Infection (n = 2)
MMF + Cytarabine (9)	66.6% (6)
	GI signs (2), Infection (1), Anemia (4)
MMF + Cyclosporin (2)	50% (1)
	GI sign (1)
Cyclosporin + Cytrabine (1)	-

MMF: mycophenolate mofetil; Cytarabine: cytosine arabinoside; and GI: gastrointestinal

## Discussion

In this study, when the survival time after each treatment was compared, the PDS group had the shortest survival time, with an MST of 42 days. Comparing the survival curves of PDS alone group and adjunctive immunosuppressants group, the latter had a significantly longer survival time. Considering that MUE is an immune mediated disease of the CNS, using an immunosuppressive dose of glucocorticoids is the primary treatment, but the result shows that the use of adjunctive immunosuppressive agents can be more beneficial than PDS alone in dogs with MUE. However, since the number of dogs in PDS monotherapy group was very small and there is possibility that other factors that cannot be considered in this study like the possibility that dogs in PDS monotherapy group died before trying adjunctive agents affected to the outcome. It is important to comprehensively check the patients’ condition, symptoms, hematologic examinations, and owner’s financial stance when adding an adjunctive immunosuppressants.

The survival times and adverse events were compared for each adjunctive immunosuppressants. The overall response rates, including CR and PR, showed a relatively good response that the CR rate of leflunomide was 16%, the PR rate was high at 69%. It was not confirmed that leflunomide can produce statistically or scientifically better outcome compared to other immunosuppressants in this study. However, considering that it have the advantage of a long half-life (21.3 h), which is longer than MMF (8 h) and there is no need to regularly visit the hospital to receive subcutaneous or intravenous injections, such as with cytarabine, leflunomide could be a good option as adjunctive treatment for MUE [22–25].

However, moderate anemia with less than 30% of PCV was confirmed in three dogs (18.7%) who used leflunomide. Anemia, confirmed with other adjunctive immunosuppressants, improved spontaneously or after supportive care, such as with gastrointestinal protectants, recovery of inflammation (acute pancreatitis, pyoderma), or reduction of the concurrent PDS. However, dogs with moderate to severe anemia (PCV 26% and 12.6%, respectively) did not show evidence of gastrointestinal bleeding or inflammation while taking leflunomide. Since anemia improved only after discontinuing leflunomide in these dogs, it is likely that the effects were due to leflunomide. It has been reported that leflunomide not only causes bone marrow suppression in humans but also causes hemolysis at high concentrations [26, 27]. Hemolytic anemia has been reported in dogs taking leflunomide (over 4 mg/kg/day) [28]. In this study, it was confirmed that reversible anemia could occur if leflunomide was administered for more than two months (even though no dogs used over 4 mg/kg/day of leflunomide). Notably, in dogs taking leflunomide, mucous membrane color and CBC should be checked regularly.

This study is the first to calculate the MST when leflunomide and PDS were used together in dogs diagnosed with MUE. However, the number of dogs treated with leflunomide was relatively small, and most of the dogs were alive at the end of the study; therefore, the estimated survival time may differ from the actual one. A follow-up study investigating the use of leflunomide over a longer period and on a larger scale is necessary.

MMF combination with PDS had longer MST of 865 days than PDS monotherapy of 42 days and 84.6% of patients showed improvement of neurological symptoms. In previous studies, MMF showed good efficacy in MUE patients, and the adverse events were relatively mild [10, 29]. There were more patients who used MMF than other groups in this study, but only 23% of patients showed adverse events, indicating that it is a well-tolerated agent to MUE dogs.

Cytarabine can cross the blood-brain barrier, therefore it has been commonly used in MUE and can be administered by constant rate infusion or subcutaneous route [6, 30–34]. The MST of cytarabine was the third longest at 754 days, but more than half of the patients (53%) showed no improvement in neurological symptoms. In a previous report, constant rate infusion of cytarabine produced longer median survival time than subcutaneous route [7]. In other reports, when cytarabine was administered subcutaneously, it was enough to reach the therapeutic target concentration in the blood [35, 36]. Because it is not clear whether cytarabine works time or concentration in dogs, so which route is better than the other is still controversial. In this study, it was difficult to compare the two routes because 15 out of 17 dogs were treated with subcutaneous route. It is considered that further studies on whether different route of administration affect survival time and treatment response are needed.

The MST of the cyclosporin + PDS treated group was significantly longer compared to the PDS monotherapy group, this result is comparable with previous report [12]. Adverse events were identified in 10 out of 18 dogs, gastrointestinal signs were most common. GI problems when using cyclosporin are relatively common, but in this study, two animals showed severe symptoms, and one dog even died due to side effects [37]. Therefore, if patients taking cyclosporin show GI problems, active supportive care such as gastrointestinal protectants and antiemetics is considered to be necessary.

Twelve dogs were in the triple therapy group, using PDS and two types of adjunctive, nine dogs were treated with the MMF + cytarabine combination, and they had a shorter MST than the double treatment groups. In the triple therapy group, 7 out of 12 dogs (58%) had adverse events, such as gastrointestinal complications or anemia, and the response rate was not higher than that of the double treatment groups. To the author’s knowledge, there is no prospective study that directly compares the efficacy and safety of using three or more immunosuppressants and using two or less immunosuppressants together. The American college of veterinary internal medicine consensus on immune-mediated hemolytic anemia treatment empirically does not recommend the concurrent use of three or more immunosuppressive drugs because there is no perceived benefit in terms of therapeutic effect or adverse events [38]. In another report, when immunosuppressive agents were used to treat canine glomerular disease, if any adjunctive immunosuppressive agent was deemed ineffective, it was recommended to change to a new drug with a different mechanism of action, rather than adding another drug [39]. It is important to recognize the increased risk of side effects and monitor the clinical signs to select an effective adjuvant immunosuppressant.

In addition, due to the limitations of this retrospective study, the follow-up period to identify adverse events was not consistent in all dogs, and the dose and treatment protocol of PDS and adjunctive immunosuppressants were not standardized. In addition, although various supportive care drugs such as anticonvulsants and analgesics were used in the patients, the duration and dosage of each patient were different. Therefore, the effect of these drugs on the patient’s survival could not be investigated. In this study, when evaluating the treatment response, the subjective views of owners and clinicians were involved, and there is a big possibility that the improvement of neurological symptoms was caused by anticonvulsants or analgesics. Also, there was no significant difference in treatment response between each agent. Therefore, it cannot be concluded which agents will produce better response in MUE patients based on the results of this study alone. Since all dogs were treated with PDS, evaluating the effect of adjunctive immunosuppressants alone is limited, and there is a possibility that any adverse events were also due to PDS. Although MUE can cause death within 2 weeks, this study included only patients who survive at least two weeks after diagnosis for comparison of treatment agents [11, 22]. This has limitations in that it biases the patients and overestimate the survival time. Therefore, large-scale prospective study in which the dosage of the drug and the interval of monitoring is required.

## Conclusions

In conclusion, the survival time of dogs with MUE can be affected depending on which immunosuppressant drugs are used, and their response to treatment. In dogs with MUE, combining PDS and adjunctive immunosuppressants such as MMF, cyclosporin, leflunomide, cytarabine can be a good option. Although there were few reports of leflunomide used in MUE, this study showed that it could be a good option as an adjunctive immunosuppressant. However periodic monitoring is necessary as possible side effects may vary for each agent. A large-scale further study with standardized doses of immunosuppressants and supportive treatment and constant monitoring interval is needed.

## Methods

### Case selection and survival time

The medical data of dogs diagnosed with MUE at Seoul National University Veterinary Medical Teaching Hospital and the VIP Animal Medical Center between January 1, 2010, and December 31, 2021, were reviewed. Patient information was reviewed using an electronic charting program (e-friends; Pet Network Veterinarian, Seoul, Republic of Korea). When additional information on the patient’s condition or survival status was needed, a telephone interview with owners was used. In the telephone interview, the owner was asked whether dogs died, the date of their death and how long they had taken the agents.

The inclusion criteria for the diagnosis of MUE were as follows: ≥ 6 months old, at least one neurological abnormality, and hyperintense lesions on T2-weighted and fluid-attenuated inversion recovery MRI. In addition, because the purpose of this study was to evaluate treatment response according to agents, patients with history of taking the agents for at least 2 weeks were included. Cases in which the infection was confirmed or where the possibility of there being a tumor was higher on MRI were excluded.

The overall survival time was calculated from the start of treatment to the death or end of study, and the survival time of each adjunctive immunosuppressants was calculated from the start of drug administration. The effects and adverse events of agents were described based on when the patient used the agents, and the survival time of each adjunctive immunosuppressants was calculated from the time the patient used the agent to death or end of study.

### Adverse events

Clinical signs, such as diarrhea, anorexia, vomiting, lethargy, and spontaneous bleeding, were considered a result of adjunctive immunosuppressants if they occurred within 2 weeks of starting treatment. CBC was conducted to evaluate myelosuppression by adjunctive immunosuppressants. Anemia was considered when the PCV was < 36%; neutropenia, when the number of neutrophils was < 3,000 cells/µL; and thrombocytopenia, when the number of platelets was < 143,000 cells/µL. The criteria for evaluating the degree of adverse events followed the Veterinary Cooperative Oncology Group Common Terminology Criteria for Adverse Events [40].

### Statistical analysis

GraphPad prism (version 9.3.1) software (GraphPad, Inc., La Jolla, CA, USA) was used for statistical analysis. The Shapiro–Wilk test was used to check whether continuous variables range of the non-parametric data. Survival time according to the patient’s characteristics and the treatment strategy was analyzed using the Kaplan–Meier method and each survival curves were compared using the log rank tests. Cases that were alive at the end of the study or died for reasons other than MUE were censored. Univariate multiple Cox proportional hazards analysis was used to determine whether there was a correlation between variables and mortality. The variables tested included sex, age, body weight at diagnosis, seizure upon admission, lesion distribution, duration of neurologic sign onset to presentation, inclusion of lesions within the brainstem, co-existence of COMS or hydrocephalus, or body weight changes during treatment. After checking whether each variable satisfies the proportional hazard assumption, two factors that did not meet (sex and brainstem lesions) were excluded, and univariate multiple Cox regression analysis was performed for the remaining factors. Differences were considered statistically significant at P < 0.05 for all analyses.

## Data Availability

All datasets used and/or analyzed during the current study are available from the corresponding author on reasonable request.

## Abbreviations

CBC: Complete blood count
CNS: Central nervous system
COMS: Caudal occipital malformation syndrome
CR: Complete response
CSF: Cerebrospinal fluid
MMF: Mycophenolate mofetil
MRI: Magnetic resonance imaging
MST: Median survival time
MUE: Meningoencephalitis of unknown etiology
NR: No response
PCV: Packed cell volume
PDS: Prednisolone
PR: Partial response
TNCC: Total nucleated cell count
